# Co-production of biodiesel and bioethanol using psychrophilic microalga *Chlamydomonas* sp. KNM0029C isolated from Arctic sea ice

**DOI:** 10.1186/s13068-020-1660-z

**Published:** 2020-02-01

**Authors:** Eun Jae Kim, Sanghee Kim, Han-Gu Choi, Se Jong Han

**Affiliations:** 1 Division of Polar Life Sciences, Korea Polar Research Institute, Incheon, 21990 Republic of Korea; 2 Department of Polar Sciences, University of Science and Technology, Incheon, 21990 Republic of Korea

**Keywords:** Arctic, Biodiesel, Bioethanol, *Chlamydomonas*, Psychrophilic microalgae

## Abstract

**Background:**

Biofuels, generated using microalgae as sustainable energy, have received a lot of attention. Microalgae can be cultivated at low cost with CO_2_ and solar energy without competition from edible crops. Psychrophilic microalgae can be a suitable feedstock to produce biofuels without the environmental constraints of low temperatures, because they can grow below 10 °C. However, there is a lack of efficient strategies using psychrophilic microalgae to produce biodiesel and bioethanol. Therefore, the current study aimed to optimize the production of biodiesel and bioethanol from Arctic *Chlamydomonas* sp. KNM0029C at low temperatures.

**Results:**

After incubation in a 20-L photobioreactor, fatty acid methyl ester (FAME) was extracted using modified FAME extraction methods, producing a maximum yield of 0.16-g FAME/g KNM0029C. Residual biomass was pretreated for bioethanol production, and the yields from different methods were compared. The highest bioethanol yield (0.22-g/g residual biomass) was obtained by pretreatment with enzyme (amyloglucosidase) after sonication. Approximately 300-mg biofuel was obtained, including 156-mg FAME biodiesel and 144-mg bioethanol per g dried cells, representing the highest recorded yield from psychrophilic microalgae.

**Conclusions:**

This is the first to attempt at utilizing biomass from psychrophilic Arctic microalga *Chlamydomonas* sp. KNM0029C for the co-production of bioethanol and biodiesel, and it yielded the highest values among reported studies using psychrophilic organisms. These results can be used as a source for the efficient biofuel production using polar microalgae.

## Background

Due to rapid industrial development, which requires the increased use of fossil fuels, concerns regarding the depletion of petroleum resources, energy security, air pollution, and global warming have led to increased global interest in developing sustainable or renewable alternative energy sources. Many nations anticipate that biofuels will soon become vital for self-sufficient energy production, as well as for decreasing the emissions of air pollutants and greenhouse gases such as carbon dioxide, nitrogen oxide, sulfur oxides, and methane [1]. Among the currently available renewable energy sources, biofuels are considered an eco-friendly and sustainable energy source, and are produced from biomass such as agricultural produce or organic waste materials [2]. Generally, vegetable and animal organic matter is thermally and chemically decomposed or fermented by microbes to produce liquid or gas fuels such as methane, ethanol, and hydrogen. Biofuels consist of bioethanol, biodiesel, biomethane, and biobutanol. First-generation biofuels used the sugars found in edible crops, but second-generation biofuels can utilize lignin, although it is highly recalcitrant, and cellulose from sources such as wood pulp [3]. The conversion of non-degradable organic compounds to fermentable sugars requires additional technical processes such as thermal, chemical, and enzymatic treatments, which increases the cost. Recently, biofuels derived from algae were developed, and have been classified as third-generation biofuels [1]. Because microalgae are not composed of lignin, it is easy to convert them to monosaccharides for ethanol production [4]. In addition, microalgae have high growth rates, efficient biofuel production rates, and a short harvesting cycle, leading to lower cost requirements than other feedstocks. Algal oil and biomass can easily be converted into diesel and gasoline. Microalgae can be cultivated anywhere, including wasteland, coast, and sea, as long as photosynthesis is possible (which requires sunlight, water, and carbon dioxide). Furthermore, they do not compete with edible crops in terms of cultivation land or space.

The Arctic and Antarctic regions are known to have the lowest temperatures on earth. Despite the extremely harsh environmental conditions (strong winds, high ultraviolet radiation exposure, dryness, and freezing temperatures), the polar regions contain a rich diversity of microalgae [5]. To survive in such severe conditions, these microalgae produce special compounds such as antifreeze proteins, polyunsaturated fatty acids, ultraviolet radiation-screening compounds, and antioxidants [6–8]. Thus, polar microalgae might be a favorable resource for the production of alternative energy sources, the synthesis of useful substances, and wastewater treatment. Microalgae isolated from the polar regions can grow efficiently even at low temperatures to produce enough biomass for biofuel synthesis [9]. Microalgae are a suitable feedstock for biodiesel production, because they contain high concentrations of lipids in the cells [10–12]. In cold regions or in winter, using biodiesel derived from microalgae could be useful owing to their high levels of unsaturated fatty acids. This high content of unsaturated fatty acids leads to a reduction in the cold filter plugging point (CFPP), which is used to assess the fluidity of biodiesel at low temperatures. In cold-climate countries (below − 10 °C), CFPP analysis assesses whether the fuel can pass through a standardized filtration device at low temperatures, because clogging at low temperatures can cause engine shutdown [13].

Despite some advantages to the use of low temperatures, attempts to use polar microalgae as a feedstock for biofuel production have been scarce. Because they grow at such low temperatures, it is difficult not only to obtain samples, but also to maintain a low-temperature environment for cultivation at the laboratory scale. Researchers who study biofuels generally use microalgae that are found in temperate or subtropical zones. Of course, in terms of microalgal biomass production at moderate temperatures, psychrotrophic (or psychrophilic) polar microalgae may be less competitive than mesophilic microalgae. This is because of the limited temperature range of polar microalgae at these temperatures; however, polar microalgae are highly active at low temperatures. This can be exploited to obtain feedstock for biofuel production using microalgae that can grow at low temperatures in areas with long winters or persistent cold weather. Previous studies analyzed the growth rate and lipid content of 184 microalgal strains isolated from the Arctic and Antarctic regions. These samples were maintained at the Korea Polar Research Institute (KOPRI). Among all candidate strains, the strain with the highest growth rate and lipid content (ideal for biofuel production) was the *Chlamydomonas* sp. KNM0029C [14]. In this study, the Arctic *Chlamydomonas* sp., a freshwater green microalga, was selected to attempt efficient production of biodiesel and bioethanol at low temperatures. We maximized the increases in biomass through optimization of the culture medium, attempted to improve yields through modified fatty acid methyl ester (FAME) extraction methods, and produced bioethanol using the residual biomass after biodiesel extraction.

## Results and discussion

### Optimal culture medium and light intensity for *Chlamydomonas* sp. KNM0029C cultivation

To maximize the concentration of KNM0029C in culture media, we optimized TAP medium through an elimination test according to the Plackett–Burman design and the Box–Behnken design method [15]. The optimal concentrations of Tris base, NH_4_Cl, MgSO_4_∙7H_2_O, CaCl_2_, K_2_HPO_4_, KH_2_PO_4_, AcOH, and trace elements were 2.42, 0.545, 0.155, 0.05, 0.029, 0.014, 1.0 (mL), and 0.077 g/L, respectively.

**Fig. 1:**
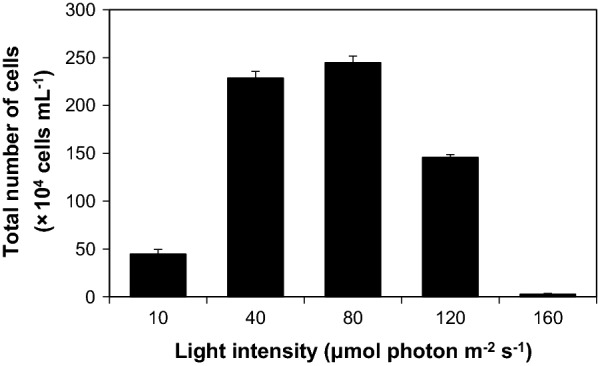
Optimal light intensity for efficient *Chlamydomonas* sp. KNM0029C growth. Data are shown as the mean ± standard deviation (SD) from two independent replicates

The effect of light on KNM0029C was investigated by exposing the samples to LED light intensities of 10, 40, 80, 120, and 160 μmol photon m^−2^s^−1^. The highest cell concentration was obtained at 80 μmol photon m^−2^s^−1^ (Fig. 1). In previously reported mesophilic strains, *Scenedesmus* sp. and *Nannochloropsis* sp. showed increased growth and biomass production at 81 and 100 μmol photon m^−2^s^−1^, respectively [16, 17]. According to a study by Heiden et al. [18], Antarctic *Fragilariopsis curta* and *Odontella weissflogii* showed maximum growth rates at 200-μmol photon m^−2^s^−1^, but their growth rates decreased at 500 μmol photon m^−2^s^−1^. At a light intensity of over 160-μmol photon m^−2^s^−1^, KNM0029C cells could not survive, because excessive light exposure causes bleaching of the cell pigment, disrupting the photosynthesis system [19].

**Fig. 2:**
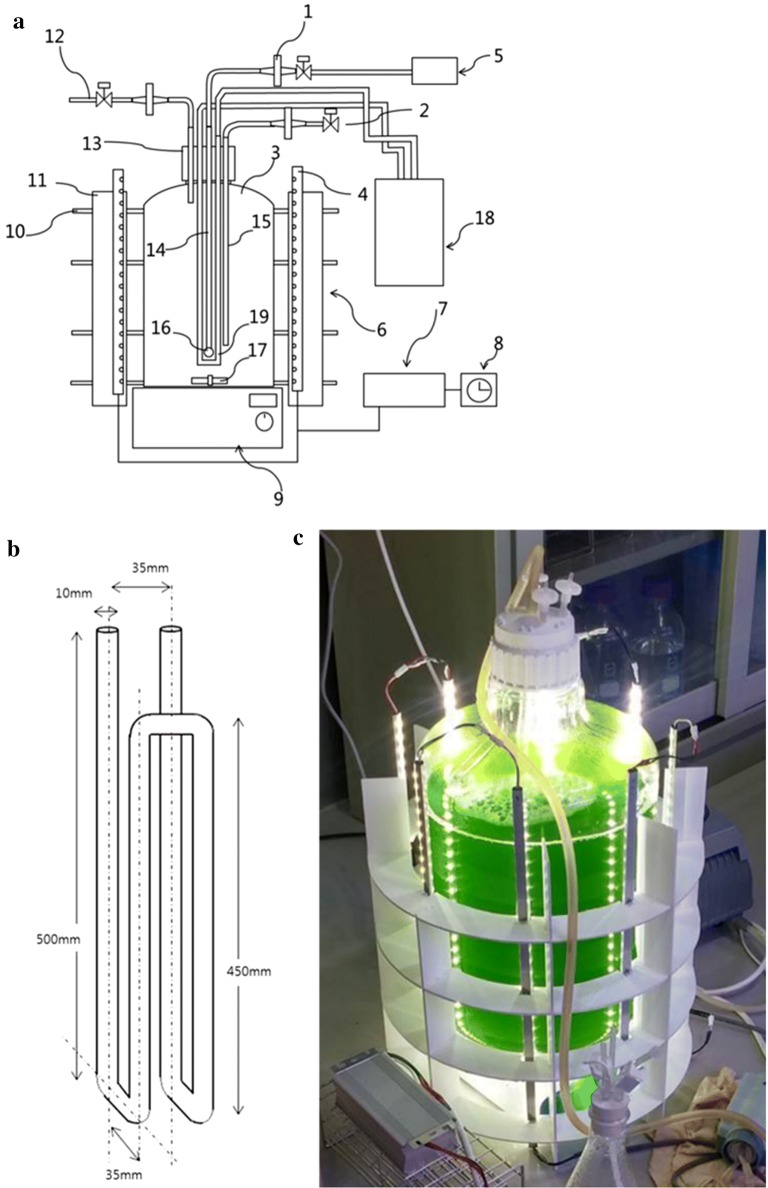
Design of the 20-L photobioreactor for Arctic *Chlamydomonas* sp. KNM0029C incubation. **a** Blueprint of the designed photobioreactor. Components are labeled as follows: 1, filter; 2, valve; 3, 20-L polycarbonate tank; 4, LED bar; 5, air supply pump; 6, LED support panel; 7, AC/DC converter; 8, timer; 9, magnetic stirrer; 10, horizontal support structures; 11, vertical support structures; 12, pressure outlet; 13, screw cap; 14, air line; 15, sampling port; 16, air stone; 17, magnetic bar; 18, cooling water circulator (chiller); 19, copper pipe for cooling. **b** Design of the copper cooling pipe. **c** Image of the 20-L photobioreactor

For KNM0029C cultivation at low temperature, a 20-L photobioreactor fitted with cooling circulator was designed (Fig. 2). Using this photobioreactor, we obtained 23-g KNM0029C after cultivation at 4 °C with 80 μmol photon m^−2^s^−1^ for 5 weeks. Growing KNM0029C under changing or high ambient temperatures is difficult; however, the low winter temperatures are beneficial for their cultivation. This is evidenced by the fact that the highest growth rates were obtained at a temperature of 4 °C [9].

### Biochemical composition of *Chlamydomonas* sp. KNM0029C

**Fig. 3:**
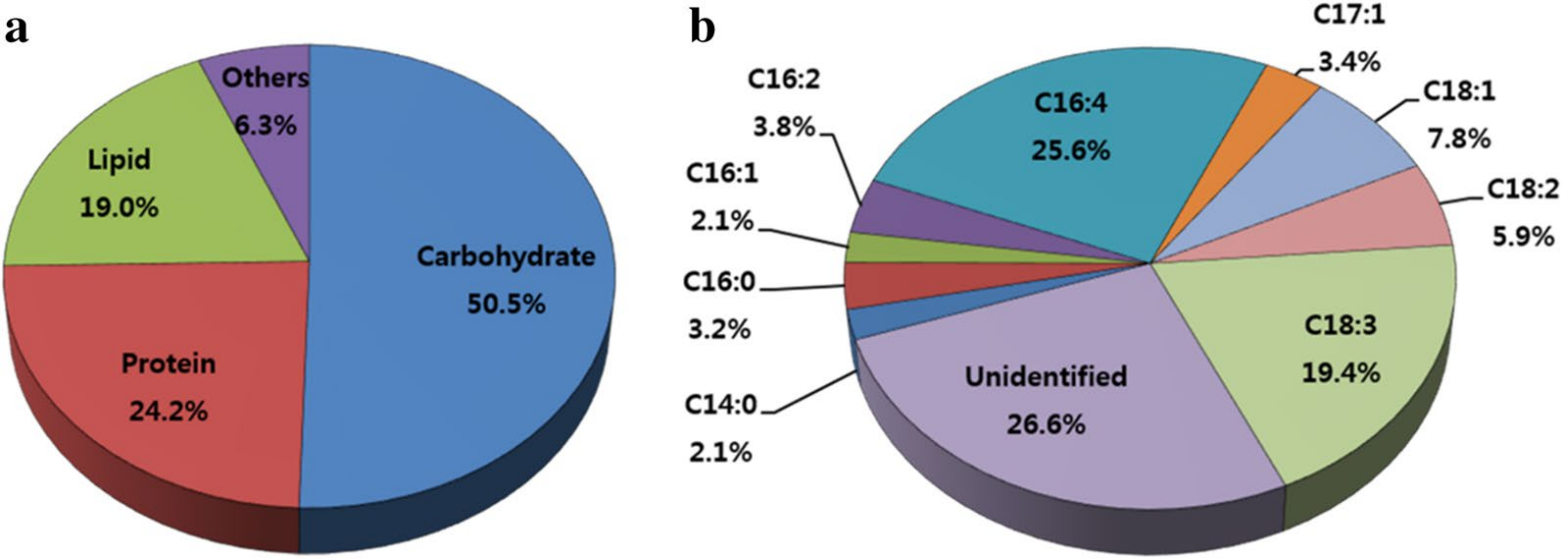
Biochemical composition of *Chlamydomonas* sp. KNM0029C at 4 °C after incubation with modified TAP at 80 μmol photon m^−2^s^−1^. **a** Contents of carbohydrates, proteins, lipids, and other components. **b** Fatty acid composition (% total fatty acid) in lipids from KNM0029C

The biochemical composition of freeze-dried KNM0029C was determined by chemical analysis. As shown in Fig. 3a, KNM0029C showed high contents of carbohydrates (50.5%) and proteins (24.2%); these components can be useful carbon and nitrogen sources for yeast fermentation to produce ethanol. The lipid content of KNM0029C was 19%, slightly lower than that of our previous study [9]. The lipid content of oleaginous microalgae is estimated to be about 13–50% [1, 20]. In our study, the lipid content of KNM0029C was relatively low; however, it was sufficient to be converted into biodiesel. Fatty acid composition analysis showed that KNM0029C contained high contents of polyunsaturated fatty acids (PUFAs) and monounsaturated fatty acids (MUFAs) such as C16:4, C18:3, and C18:1 at 4 °C (Fig. 3b). A high content of unsaturated fatty acids is known to reduce the CFPP, indicating that the biodiesel will be safe for use at low temperatures. As a feedstock for biofuel production, the carbohydrate content of KNM0029C was 50.5%, indicating that it was suitable for ethanol fermentation (Fig. 3a). In a previous study, the total carbohydrate content of *Chlamydomonas* species was reported to include mainly starch (43.6% dry cell mass), and the most predominant monomeric sugar was glucose, which could be rapidly fermented by *S. cerevisiae* [21].

### Comparison of FAME extraction methods for biodiesel production

**Fig. 4:**
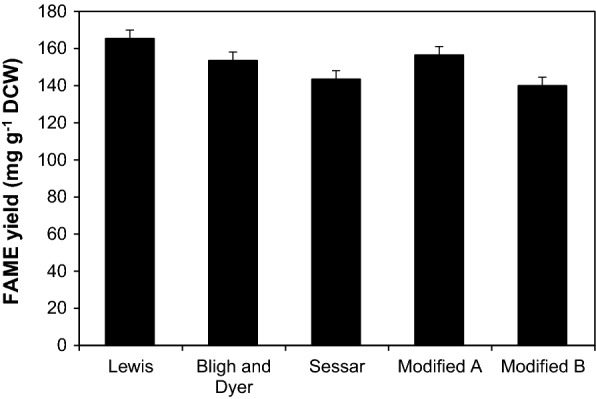
Comparison of fatty acid methyl ester (FAME) yields from biomass of KNM0029C using five different methods. Data are expressed as the mean ± SD from two independent replicates. *DCW* dry cell weight

Lewis’s method, Bligh and Dyer’s (B&D) method, and Sasser’s method were used for converting microalgal lipids to biodiesel [22–24]. These methods all used freeze-dried samples, while our modified methods A and B used wet biomass. The dehydrated biomass was useful for accurate mass measurement, and the extraction rate of crude lipids can be increased by removing moisture [25]. On the other hand, using wet biomass can reduce the time and cost associated with the process of freeze-drying [26]. To improve the extraction efficiency of the modified methods A and B, the wet biomass was sonicated (Table 1). The chloroform/methanol-based extractions (Lewis’s method and B&D’s method) resulted in slightly higher total fatty acid recovery than the methanol/hexane/methyl tert-butyl ether-based method (Sasser’s method). The recovery of FAME was highest using Lewis’s method with freeze-dried samples, followed by that using modified method A, which utilized wet biomass (Fig. 4). In a previous study, Burja et al. [27] reported that most lipids from *Thraustochytrium* sp. were recovered by the B&D-based method, but not the Lewis’s method, that recovered much less. Tommasi et al. [28] reported a similar result, where the B&D method recovered most of the fatty acids from *Phaeodactylum tricornutum*. In contrast, Cavonius et al. [29] reported that the Lewis’s method showed higher fatty acid recovery from *Nannochloropsis oculata* and *Isochrysis galbana*, than B&D’s method. Cavonius et al. and Martins et al. [29, 30] suggested that the differences in the algal cell walls could influence the efficiency of various extraction methods. Therefore, it is necessary to select a suitable FAME extraction method for algal species. The FAME yields of Lewis’s method and modified method A were 165.4 and 156.5 mg/g dry cell weight, respectively (Fig. 4). These results suggested that FAME could be extracted from wet biomass without a significant reduction in yield (− 5.4%). Modified method A showed a greater yield than the methods of B&D and Sasser. These results show that using wet biomass reduced the time and energy required for freeze-drying while still producing acceptable yields.

**Table 1:** Comparison of fatty acid methyl ester (FAME) extraction methods for biodiesel production from *Chlamydomonas* sp. KNM0029C

Method	Pretreatment	Lipid extraction	Catalyst; condition	Solvent	References
Lewis	Freeze-drying biomass (100 mg)	Methanol/chloroform (10:1)	HCl; 90 °C, 2 h	Hexane/chloroform (1:1)	[22]
Bligh & Dyer	Freeze-drying biomass (100 mg)	Methanol/chloroform (2:1)	HCl; 90 °C, 2 h	Chloroform	[23]
Sasser	Freeze-drying biomass (100 mg)	Methanol/hexane/methyl tert-butyl ether (2:1:1)	HCl: 80 °C, 10 min	Hexane/methyl tert-butyl ether (1:1)	[24]
Modified FAME extraction A	Wet biomass (100 mg DCW), sonication treatment	Methanol/chloroform (2:1)	HCl; 90 °C, 1 h	Chloroform	This study
Modified FAME extraction B	Wet biomass (100 mg DCW), sonication treatment	Methanol/chloroform (10:1)	HCl; 90 °C, 1 h	Hexane/chloroform (1:1)	This study

*DCW* dry cell weight

### Pretreatment for bioethanol production

**Fig. 5:**
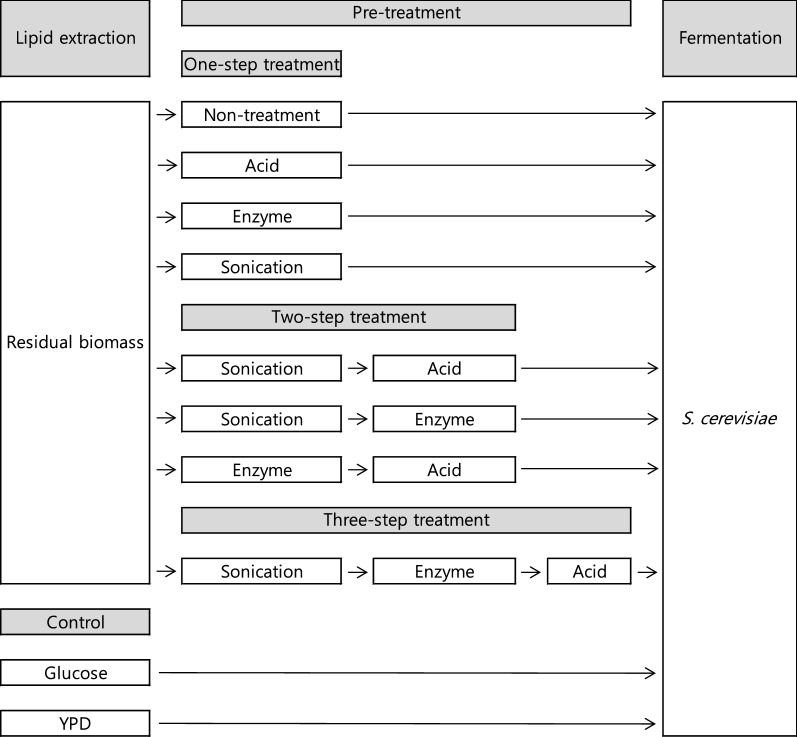
Schematic illustration of the different hydrolysis methods used on the lipid-extracted polar microalgal biomass for bioethanol production. *YPD* yeast extract peptone dextrose medium

**Fig. 6:**
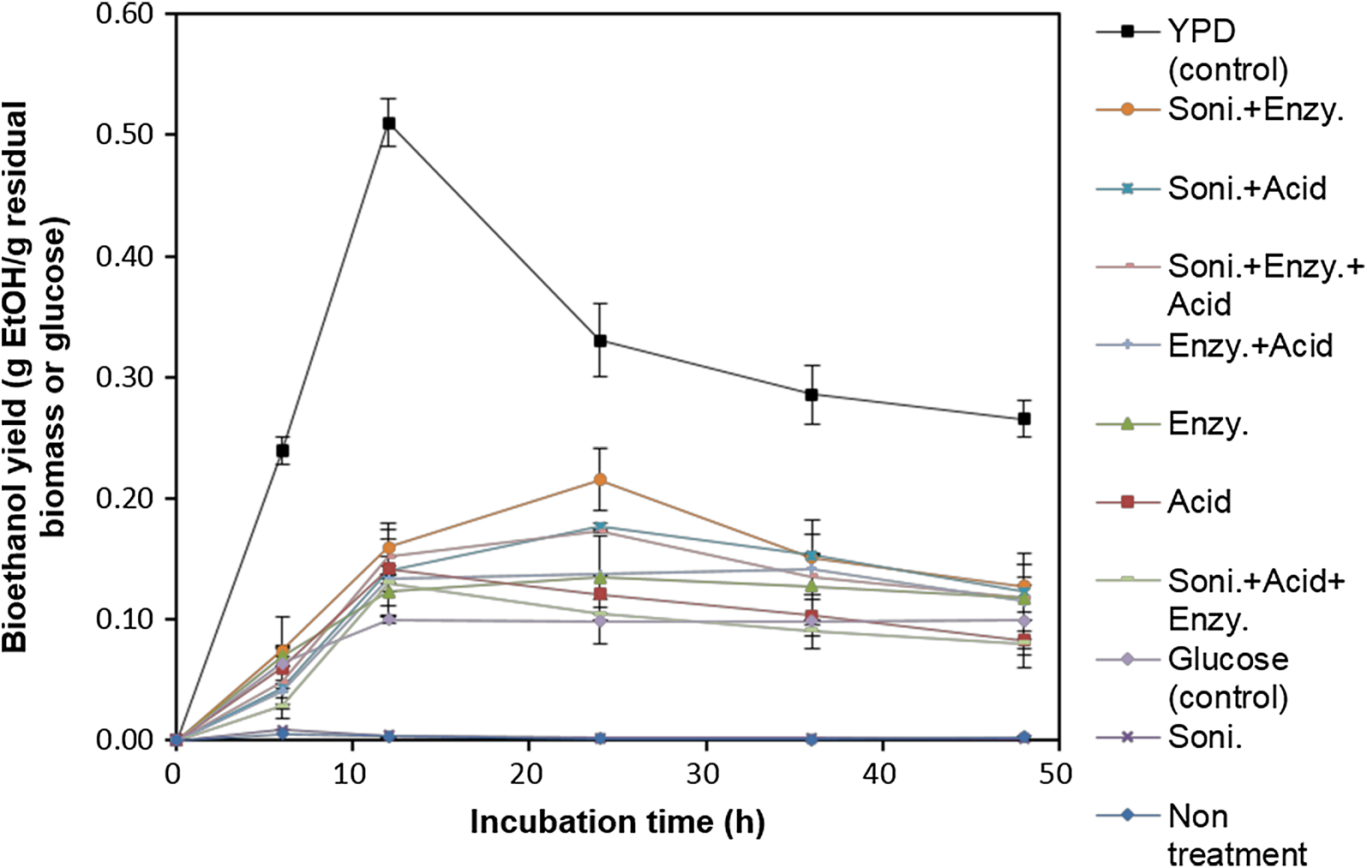
Effects of different hydrolysis methods on bioethanol yield in polar microalgal biomass. Sonication (Soni.), acid, and amyloglucosidase enzyme (Enzy.) were used for pretreatment, and the pretreated biomass was fermented by *Saccharomyces cerevisiae* to produce ethanol. Data are shown as the mean ± SD from three independent experiments. *YPD* yeast extract peptone dextrose

**Fig. 7:**
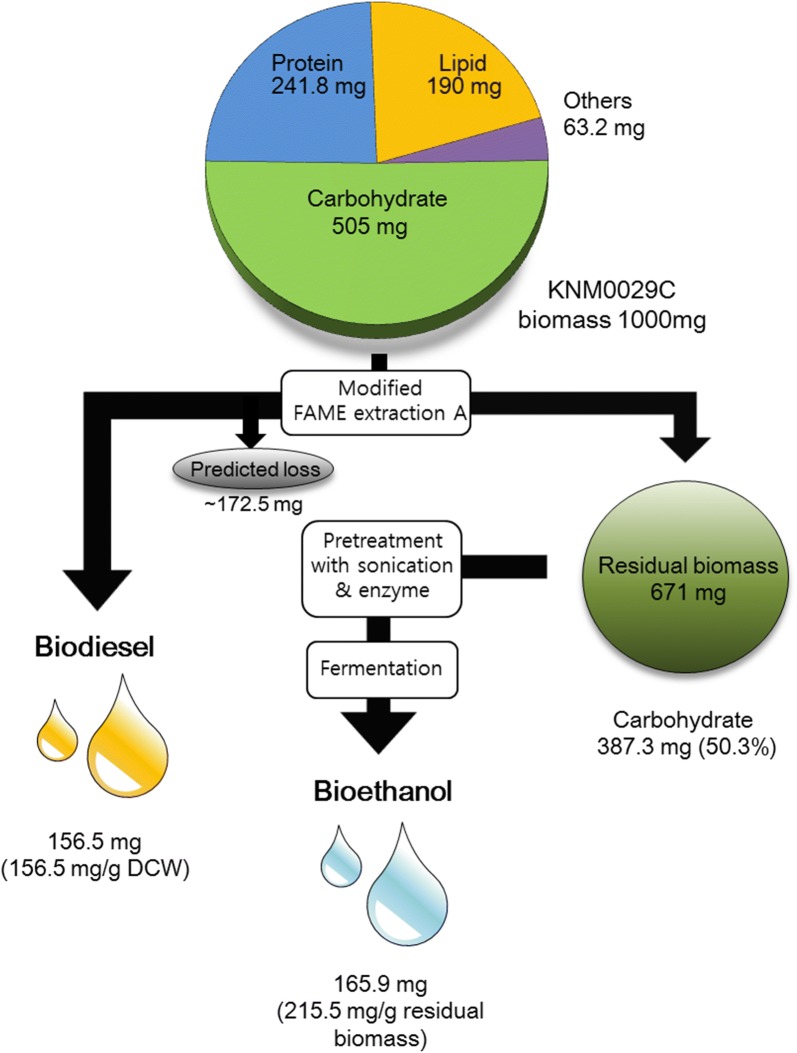
Conversion of biomass of Arctic *Chlamydomonas* sp. KNM0029C into biofuels

After lipids are extracted from microalgae to make biodiesel, the residual biomass, which is generated as a byproduct, can be fermented with yeast to produce ethanol [21]. In this study, FAME was extracted from KNM0029C and the residual biomass was used to produce bioethanol. After FAME extraction, the carbohydrate content was approximately 50.3% (w/w) of the residual biomass (Table 2). Thus, there was a reduction (172.5 mg) in carbohydrate content due to the lipid extraction process. In a previous study by Kim et al. [34], the strain with the highest carbohydrate content (60% dry cell weight) was *Chlamydomonas reinhardtii* IAMC-238. The biochemical content of the residual biomass of KNM0029C included mainly carbohydrates (50.3%), most of which was starch. The enzyme AMG 300L was selected to decompose the starch into monosaccharide, and showed high efficiency at 0.4 mL/g dried biomass in the saccharification reaction [40]. The high carbohydrate content of KNM0029C has the advantage of providing the carbon source needed for ethanol fermentation (Table 2). Pretreatment using residual biomass was performed, as shown in Fig. 5 and consisted of one-step, two-step, and three-step processes. The one-step processes included either physical (sonication), chemical (acid), or biochemical (enzyme) treatments; the two-step processes included physical + chemical treatments, physical + biochemical treatments, or biochemical + chemical treatments; and the three-step process included a combination of physical, biochemical, and chemical treatments (Fig. 5). After pretreatment, samples were sterilized and inoculated with *S. cerevisiae*, and the ethanol yield was determined by GC analysis. The sample with the highest ethanol yield was the YPD control sample after 12 h of culture, with a yield of 0.51 g/g of glucose; this was consistent with the theoretical value of 0.51 g ethanol/g glucose (Fig. 6). According to a recent study by Della-Bianca et al. [41], about 0.45 g ethanol/g glucose was produced in YPD medium. In another study, 0.48 g ethanol/g glucose was produced in YPD medium [42]. However, a single glucose, which was another control, produced approximately 0.1 g ethanol/g glucose in 12–48 h of culture. It was presumed that the yield depends on the presence or absence of a nitrogen source [43]. The method showing the highest ethanol yield among the pretreated residual biomass samples was the two-step physical + biochemical process, which was fermented after sonication and treatment with AMG 300L (Fig. 6) and produced a yield of 0.22 g ethanol/g residual biomass at 24 h. This value was higher than that previously obtained (0.16 g/g residual biomass) by fermenting residual *Chlorella* sp. KR-1, as reported by Lee et al. [44]. The physical + chemical and three-step methods produced 0.18- and 0.17-g ethanol/g residual biomass, respectively (Fig. 6). The use of combined physical, chemical, and biological treatments to convert carbohydrates to monosaccharides was inefficient overall. Although the combined treatment has been shown to enhance hydrolysis from feedstock, the hydrolyzed sugars may be fructose and galactose rather than glucose, which is the preferred carbon source for fermentation using *S. cerevisiae* [3]. Based on this result, we conclude that the three-step pretreatment process was unnecessary for bioethanol production from residual KNM0029C biomass. To minimize costs, the use of acid instead of enzymes is preferable for ethanol production. Samples pretreated with acid produced 0.18-g ethanol/g residual biomass. This yield was slightly lower than that of enzyme-pretreated samples; however, it was estimated that the economic efficiency was higher than that of enzyme treatment because of the cheap price of acids (Table 3). In a study by Lee et al. [47], 0.40-g ethanol/g dried biomass was produced from *Chlorella vulgaris* hydrolyzed by pretreatment with enzyme and acid. Furthermore, Nguyen et al. obtained a yield of 0.29-g ethanol/g dried biomass from *C. reinhardtii* hydrolyzed by acid treatment (Table 3). However, both of these two studies used non-residual biomass. In studies using residual biomass, Lee et al. [40] and Lee et al. [44] obtained yields of 0.14-g and 0.16-g ethanol/g dried biomass from residual *Dunaliella tertiolecta* and *Chlorella* sp. KR-1, respectively, after pretreatment with enzyme and acid (Table 3). In the current study, the residual KNM0029C biomass after lipid extraction treated through a two-step sonication and enzyme treatment process, and then fermented with yeast, yielded 0.22-g ethanol/g residual biomass. To our knowledge, this value is the highest obtained for fermentation of ethanol from residual biomass after extracting lipids from psychrophilic microalgae. We used Arctic *Chlamydomonas* sp. KNM0029C as a feedstock for biofuels, and as shown in Fig. 7, 156.5-mg biodiesel and 144.6-mg bioethanol were produced from 1000-mg biomass. Despite the ongoing efforts to develop microalgae as the source organisms for biofuel production, the economic feasibility of this strategy is still low. Attempts to identify and exploit microalgae that can grow at low temperatures will help to improve the productivity and cost effectiveness, by overcoming the environmental and seasonal impediments [9, 51]. Unlike previous studies, which investigated biofuel production using mesophilic microalgae, this study is the first to attempt to produce both bioethanol and biodiesel from psychrophilic Arctic microalgae. These findings could be valuable to increase production efficiency without seasonal effects leading to poor microalgal growth at low temperatures.

**Table 2:** Carbohydrate content of green microalgae

Microalgal species	Carbohydrate content (%)	References
*Chlorella vulgaris* IAM C-534	37.0	[31]
*C. vulgaris*	55.0	[2]
*Nannochloropsis* sp.	32.1	[32]
*Desmodesmus* spp.	41.0	[33]
*Chlamydomonas reinhardtii* UTEX 90	59.7	[21]
*C. reinhardtii* IAM C-238	60.0	[34]
*Scenedesmus acutiformis* TISTR8495	16.4	[35]
*S. obliquus* CNW-N	51.8	[36]
*Chlorococum* sp. TISTR8583	26.0	[37]
*Chlorococum* sp.	32.5	[38]
*Tetraselmis* sp. CS-362	26.0	[39]
*Chlamydomonas* sp. KNM0029C	50.5 (50.3)^a^	This study

^a^Carbohydrate content of residual biomass after lipid extraction

**Table 3:** Comparison of the ethanol content of *Chlamydomonas* sp. KNM0029C and those of other strains with different pretreatment protocols

Algal feedstock	Pretreatment	Fermenting organism	Content (%) (g EtOH/g dry mass)	References
*Gracilaria salicornia* (Macro-)	H_2_SO_4_ at 120 °C, cellulase	*Escherichia coli* KO11	7.9	[45]
*Spirogyra* sp. (filamentous)	Cellulose and amylase	*Saccharomyces cerevisiae*	8	[46]
*Chlamydomonas reinhardtii*	Amylase and glucoamylase	*S. cerevisiae* S288C	23.5	[21]
*Chlorella vulgaris*	H_2_SO_4_ at 110 °C, cellulose and β-glucosidase	*E. coli* SJL2526	40	[47]
*C. reinhardtii*	H_2_SO_4_ at 110 °C	*S. cerevisiae* S288C	29.1	[48]
*Chlorella minutissima*	H_2_SO_4_ at 100 °C	*S. cerevisiae*	18.5	[49]
*Chlorella*	H_2_SO_4_ at 120 °C, α-amylase	*S. cerevisiae*	28.1	[50]
Residual *Dunaliella tertiolecta*	Amyloglucosidase	*S. cerevisiae*	14	[40]
Residual *Chlorella* sp. KR-1	HCl at 121 °C	*S. cerevisiae*	16	[44]
Residual *Chlamydomonas* sp. KNM0029C	Sonication and amylase	*S. cerevisiae*	21.6	This study
Residual *Chlamydomonas* sp. KNM0029C	Sonication and HCl at 121 °C	*S. cerevisiae*	17.6	This study
Residual *Chlamydomonas* sp. KNM0029C	Sonication and amylase, HCl at 121 °C	*S. cerevisiae*	17.3	This study

## Conclusions

To obtain efficient biofuel production at low temperatures, we produced FAME biodiesel and bioethanol, using the psychrophilic *Chlamydomonas* sp. KNM0029C. The biochemical contents of freeze-dried KNM0029C were determined to be 50.5% carbohydrate, 19% lipid, and 24.2% protein. When modified method A was used for FAME extraction, the obtained yield was 156.5-mg/g dry cell weight. After biodiesel extraction, bioethanol was produced from the residual biomass. The sonication and amyloglucosidase treatment method produced the highest reported ethanol yield of 0.22-g ethanol/g residual KNM0029C biomass. Overall, we obtained 300 mg of biofuel per g dried cells, which was the highest yield from psychrophilic microalgae to date. The microalga *Chlamydomonas* sp. KNM0029C was successfully used as a feedstock for biofuels, and these results can be utilized for the efficient production of biodiesel and bioethanol.

## Methods

### Isolation and purification of microalgal strains

The green microalga *Chlamydomonas* sp. KNM0029C (formerly known as ArM0029C) was collected from Arctic sea ice near the Dasan station in Ny-Ålesund, Norway (78°55′ N, 11°56′ E). Samples were cultured in Bold’s basal medium (BBM) as previously described at 2–3 °C under white light-emitting diodes (LED) [52]. For the isolation of a single strain, cultures were serially diluted, and the diluents were plated on BBM agar plates. Thereafter, a single green colony was picked and cultured in BBM broth.

### Culture conditions

Due to the previously shown high growth rates in the modified Tris-Acetate-Phosphate (TAP) medium at 4 °C [9, 15], we used this medium to culture *Chlamydomonas* sp. KNM0029C. To determine the optimal light intensity, 2.5 × 10^5^ cells mL^−1^ were inoculated in modified TAP medium and cell growth was measured under static conditions at 4 °C under various white fluorescent lights (10-, 40-, 80-, 120-, and 160-μmol photon m^−2^s^−1^) on a 16:8-h light:dark cycle. The microalgal samples obtained from two independent replicates were counted using a hemocytometer with an optical microscope (Zeiss Axio Imager 2; Zeiss, Oberkochen, Germany).

A 20-L photobioreactor made of transparent carboy polycarbonate (Nalgene 2261-0050, Thermo Fisher Scientific, Waltham, MA, USA) was used. A copper pipe was connected to the cooling circulator (RW-0525G, Jeio Tech, Korea) and attached to the cap plug (as shown in Fig. 2). In addition, an air injection line was installed. By controlling the temperature of the cooling circulator, the internal medium temperature of photobioreactor was maintained at 4 °C. The air was supplied by an air pump (BT-A65, Philgreen, Korea) at 4 L min^−1^. Eight LED bars (LG Innotek Co., Korea) with AC/DC converter (IDF100CV-S12V1, IDF Co., Korea) were installed to a stand, constructed with 4-mm polyvinyl chloride (PVC) foam board, to supply light. With the help of the cooling circulator, the temperature of the culture medium was adjusted to 4 °C, while the LED bars were set to provide a light intensity of 80-μmol photon m^−2^s^−1^. The medium was mixed using a magnetic stirrer (MS 200, Misung Scientific Co., Korea) at a stirring rate of 100 rpm.

### Biochemical composition analysis

#### Lipids

Crude lipids were extracted from the disrupted biomass (wet weight). Then, 15-mL CHCl_3_:CH_3_OH (1:2) was added, and the solution was ultra-sonicated for 10 min (VCX 750; Sonics, Newtown, CT, USA) at 4 °C with pulsing (35%, 20 ± 1 W, pulse on/off = 1 s/5 s), and lipids were extracted with inverting for 1 h at room temperature. After lipid extraction, the residual biomass was dried at 60 °C overnight.

#### Carbohydrates

The carbohydrate content was determined using phenol–sulfuric acid. The biomass sample (20 mg) was resuspended in 20-mL distilled water and diluted ten-fold. Next, 500-μL diluted sample was transferred to a 15-mL centrifuge tube and 0.5-mL phenol (5%, w/v) was added, followed by reaction with 2.5-mL concentrated sulfuric acid (72%, w/w). The mixtures were vortexed and incubated for 20 min at room temperature. The absorbance at 470 nm was measured using a UV–Vis spectrophotometer (Ultraspec 3300 pro, Amersham Biosciences, USA). The absorbance values were analyzed relative to a standard curve based on glucose.

#### Proteins

Protein content was analyzed using the Bradford method [53]. Freeze-dried cells were disrupted by sonication, and the solution was stained with Bradford reagent (Sigma-Aldrich, St. Louis, MO, USA).

### Conversion to FAME for biodiesel production

Algal lipids were extracted from 100-mg freeze-dried KNM0029C sample and 3-mL CHCl_3_:CH_3_OH (1:2, v/v) was added. Tubes were vortexed for 1 min, followed by the addition of 1-mL distilled water to separate the organic phase. Thereafter, 1-mL CHCl_3_ was added and samples were vortexed for 30 s. Tubes were centrifuged at 2500×*g* for 10 min to recover the CHCl_3_ phase, which was transferred to a preweighed glass vial. The organic solvent was removed using an evaporator, and lipids were weighed after drying at 60 °C for 2 days. To convert the lipids to FAME, dried lipids were treated with 1-mL saponification reagent (7.5-M NaOH:CH_3_OH, 1:1, v/v) at 100 °C for 30 min; thereafter, the samples were incubated on ice for 10 min, and 2-mL CH_3_OH:6-N HCl (1:1, v/v) was added and incubated at 80 °C for 10 min. Reactions were then performed with 1.5-mL hexane:methyl tert-butyl ether (1:1, v/v) for 10 min. The lower aqueous phase was discarded and 3-mL 0.5-M NaOH was added to the organic phase. The organic phase of the top layer was collected in gas chromatography (GC) vials for GC analysis. Organic phase FAMEs were analyzed by gas chromatography (YL-6100GC, Young Lin Science, Korea) with a flame-ionized detector (FID) and Omegawax 250 capillary column (30 m × 0.25 mm × 0.25 μm, Supelco, USA). FAME analysis was performed under the following conditions: constant flow mode (3 mL min^−1^); temperature, maintained at 50 °C for 2 min, and then 4 °C min^−1^ to 220 °C (for 15 min); and detector temperature (260 °C). FAME components were identified by the Supelco 37 Component FAME Mix (Sigma-Aldrich, St Louis, MO, USA). FAME was quantified against methyl tricosanoate C23:0 (Sigma-Aldrich, St Louis, MO, USA) as the internal standard [9].

### Comparison of five FAME extraction methods

#### Lewis’s extraction

Freeze-dried samples (100 mg) were incubated at 90 °C for 2 h with 15-mL CH_3_OH:HCl:CHCl_3_ (10:1:1). Then, 5-mL distilled water was added and the FAMEs were extracted by adding 10-mL hexane:CHCl_3_ (4:1). After the tubes were vortexed for 2 min, the top layer was recovered [22].

#### Bligh and Dyer’s extraction

Distilled water (400 μL) was added to freeze-dried sample (100 mg). Next, 1.5-mL CHCl_3_:CH_3_OH (1:2) was added and the sample was vortexed for 2 min. Thereafter, 100-μL CHCl_3_ was added, the sample was vortexed for 30 s, 500-μL distilled water was added to separate the two phases, and the sample was vortexed for a further 30 s. The sample was then centrifuged at 2500×*g* for 10 min, the aqueous phase was removed, and the organic phase was recovered. Next, 2-mL HCl was added, and the sample was incubated at 90 °C for 2 h. Finally, 5-mL distilled water was added, and FAMEs were extracted by adding 10-mL CHCl_3_. After the tubes were vortexed for 2 min, the top layer was recovered [23].

#### Sasser’s extraction

Algal fatty acids were extracted from 100-mg freeze-dried samples as described by Sasser [24]. Saponification was performed using 10-mL saponification reagent (7.5 M NaOH:CH_3_OH, 1:1) at 100 °C for 30 min; thereafter, the samples were incubated on ice for 10 min. Methylation was performed by incubating samples with 20-mL methylation reagent (CH_3_OH:6 N HCl, 1:1) at 80 °C for 10 min. Reactions were then performed by incubating samples with 12.5-mL hexane:methyl tert-butyl ether (1:1 v/v) for 10 min. The lower aqueous phase was discarded, and 30-mL 0.5-M NaOH was added to the organic phase. The top layer was collected in GC vials.

#### Modified FAME extraction A

To prepare the wet biomass, 100-mg freeze-dried sample was added to 1-mL distilled water and vortexed for 10 min. The sample was centrifuged at 2500×*g* for 10 min, and the clear aqueous phase was removed. Then, 1.5-mL CHCl_3_:CH_3_OH (1:2) was added and the sample was ultra-sonicated for 10 min (VCX 750, Sonics) at 4 °C with pulsing (35%, 20 ± 1 W, pulse on/off = 1 s/5 s), followed by the addition of 100-μL chloroform. The sample was vortexed for 30 s, 500-μL distilled water was added to separate the two phases, and the sample was vortexed for another 30 s. After the sample was centrifuged at 2500×*g* for 10 min, the aqueous phase was removed, and the organic phase was recovered. Next, 2-mL HCl was added and the sample was incubated at 90 °C for 2 h. Finally, 5-mL distilled water was added, and the FAMEs were extracted by adding 10 mL chloroform. After the tubes were vortexed for 2 min, the top layer was recovered.

#### Modified FAME extraction B

Wet biomass was prepared from 100-mg freeze-dried sample as described above, and was added to 15-mL CH_3_OH:CHCl_3_ (10:1) and ultra-sonicated for 10 min. Next, 2-mL HCl was added and the sample was incubated at 90 °C for 2 h. Then, 5-mL distilled water was added and the FAMEs were extracted by adding 10 mL hexane:CHCl_3_ (1:1). After the tubes were vortexed for 2 min, the top layer was recovered.

### Pretreatment of residual biomass for bioethanol production

Commercial amyloglucosidase (AMG 300L; EC 3.2.1.3) was purchased from Sigma-Aldrich. The enzyme activity of AMG 300L was 300 amyloglucosidase units (AGU)/mL. All solvents were purchased from Duksan Chemical Co. (Ansan-si, Korea). Glucose was purchased from Sigma-Aldrich. Peptone and dextrose were purchased from Merck (Darmstadt, Germany). Yeast extract was purchased from Samchun Chemical Co. (Seoul, Korea). The three pretreatment methods described above were performed in eight different combinations. Glucose (200 mg) and yeast extract peptone dextrose (YPD; glucose: 200 mg, yeast extract: 80 mg, and peptone: 160 mg) in 8 mL of distilled water were used as a control.

#### Sonication treatment

For sonication treatment, 400-mg residual biomass was added to 8-mL distilled water and ultra-sonicated for 10 min (VCX 750, Sonics) at 4 °C with pulsing (35%, 20 ± 1 W, pulse on/off = 1 s/5 s).

#### Acid treatment

For acid treatment, 400-mg residual biomass was added to 8 mL of distilled water, treated with 331-μL HCl (37%, w/w), and autoclaved at 121 °C for 15 min.

#### Enzymatic treatment

Enzymatic treatment was performed with 400-mg residual biomass in 8 mL of distilled water at 55 °C and pH 5.5. Samples were incubated with 160-μL AMG 300L for 60 min.

### Ethanol production using hydrolysates of the residual biomass

All pretreated samples were autoclaved at 121 °C for 15 min after adjusting the pH to 6.5 using 3-M NaOH or 3-M HCl. *Saccharomyces cerevisiae* was used for ethanol fermentation of the hydrolysate products from the residual biomass. For seed culture, *S. cerevisiae* was cultured in 15-mL YPD medium at 30 °C and 150 rpm for 24 h. The composition of the YPD medium was as follows: yeast extract, 10 g/L; peptone, 20 g/L; dextrose, 20 g/L. For ethanol fermentation, 800-μL seed culture was inoculated in 8-mL saccharified sample in a 50-mL culture tube at 30 °C and 150 rpm. Sampling was performed at 0, 6, 12, 24, 36, and 48 h after inoculation.

### Analysis of bioethanol content

To quantify the production of bioethanol, 1-μL filtered sample was subjected to GC analysis; the peak area was compared with the standard (10%, 1%, 0.1% ethanol) to determine the concentration of ethanol using the Omegawax capillary column (I.D. 30 m × 0.32 mm × 0.25 μm; Supelco, Sigma-Aldrich) with nitrogen as the carrier gas. GC was performed at a flow rate of 2 mL/min, with the injector temperature maintained at 220 °C, a split ratio of 20:1, and a GC-FID temperature of 240 °C. The oven temperature was maintained at 60 °C for 10 min.

## Data Availability

The data sets used and/or analyzed in this study are available from the corresponding author upon reasonable request.

## Abbreviations

AGU: Amyloglucosidase units
AMG: Amyloglucosidase
B&D method: Bligh and Dyer’s method
BBM: Bold’s basal medium
CFPP: Cold filter plugging point
FAME: Fatty acid methyl ester
FID: Flame ionization detector
GC: Gas chromatography
LED: Light-emitting diodes
MUFAs: Monounsaturated fatty acids
PUFAs: Polyunsaturated fatty acids
PVC: Polyvinyl chloride
RT: Retention time
TAP: Tris–acetate-phosphate
UV: Ultraviolet
YPD: Yeast extract–peptone–dextrose
